# Comparison of metrics for assessing face washing behaviour for trachoma control

**DOI:** 10.1371/journal.pntd.0012399

**Published:** 2024-08-14

**Authors:** Katie Greenland, Claire Collin, Edao Sinba Etu, Meseret Guye, Demitu Hika, David Macleod, Wolf-Peter Schmidt, Oumer Shafi Abdurahman, Anna Last, Matthew J. Burton

**Affiliations:** 1 Department for Disease Control, London School of Hygiene & Tropical Medicine (LSHTM), London, United Kingdom; 2 Berhan Public Health & Eye Care Consultancy, Addis Ababa, Ethiopia; 3 The Fred Hollows Foundation Ethiopia, Addis Ababa, Ethiopia; 4 International Statistics and Epidemiology Group, LSHTM, London, United Kingdom; 5 International Centre for Eye Health, Clinical Research Department, LSHTM, London, United Kingdom; 6 Clinical Research Department, London School of Hygiene & Tropical Medicine (LSHTM); 7 National Institute for Health Research Biomedical Research Centre for Ophthalmology at Moorfields Eye Hospital NHS Foundation Trust and UCL Institute of Ophthalmology, London, United Kingdom; Medical University of Vienna, AUSTRIA

## Abstract

There is currently no single, easy-to-use, reliable indicator to assess whether a face has been washed with soap in the context of trachoma elimination. This study aimed to compare survey report, script-based pictorial recall and facial cleanliness indicators as alternatives to structured observation for measuring face washing behaviour. This method validation study was nested in the Stronger-SAFE trial, Oromia Region, Ethiopia. Structured observation was conducted in randomly selected households for three hours from dawn. The primary caregiver in each household participated in a survey to capture (self)-reported behaviour and/or script-based pictorial recall, a routine-based diary activity to covertly capture information on face washing behaviour of themself and any children aged 1–12. Children 4–12 years old directly participated in the survey and pictorial recall in a subset of households. The facial cleanliness of children aged 1–12 was assessed qualitatively and using the quantitative Personal Hygiene Assessment Tool (qPHAT). Prevalence estimates, sensitivity, specificity and predictive values were computed for each behavioural indicator with observation data as the gold standard. The appropriateness of script-based pictorial recall was assessed using baseline and 3-month follow-up data. Baseline data were collected from 204 households in 68 clusters. Survey estimates of face washing and face washing with soap among caregivers and children were 32% to 60% and 5% to 31% higher than observed behaviour, respectively. Face washing prevalence estimates from pictorial recall were lower than survey estimates and comparable with observations for some face washing with soap indicators (0.3% to 13% higher than observations). Specificity of pictorial recall indicators was high (85% to 99%), but the sensitivity was low (0% to 67%), resulting in a low positive predictive value for all indicators. Both qualitative facial cleanliness indicators and qPHAT scores were poorly correlated with observed face washing earlier that morning. Pictorial recall overestimated face washing with soap among both caregivers and children following intervention delivery but not at baseline. Survey (self)-reported data on face washing is highly inaccurate. Script-based pictorial recall does not correctly classify those who wash their face with soap, and is subject to differential bias following intervention exposure, and facial cleanliness is a poor indicator of recent face washing in settings where faces become rapidly dirty again after washing. Alternatives to structured observation cannot be recommended to monitor the effectiveness of face washing interventions in community settings.

**Trial Registration** ISRCTN registry ISRCTN40760473, https://doi.org/10.1186/ISRCTN40760473.

## Author summary

This study aimed to identify easy-to-use indicators for face washing behaviour to improve the evaluation of behaviour change interventions in the context of trachoma elimination. The study compared reported behaviour, script-based pictorial recall (a routine-based diary activity to covertly capture information on face washing behaviour) and facial cleanliness indicators with direct observation of face washing. Facial cleanliness assessment and qPHAT scores were poorly correlated with observed behaviour. Caution should be applied before using facial cleanliness metrics as a proxy for face washing. Survey report produced much higher estimates of face washing and face washing with soap than were observed. Pictorial recall overestimated face washing with soap in the intervention arm following intervention exposure, suggesting the metric reflects knowledge rather than behaviour. None of the tested alternatives to structured observation can be recommended to monitor the effectiveness of face washing interventions in community settings.

## Background

### Face washing for trachoma control

Trachoma, blinding eye condition, is caused by the bacterium *Chlamydia trachomatis* (Ct). Repeated infections result in painful sight loss in the absence of intervention [1]. These more serious consequences of trachoma predominantly affect women and are estimated to total $8 billion annually in lost productivity [2].

Ocular *C*. *trachomatis* is thought to spread from eye-to-eye directly and via fingers, on fomites such as towels and bedding, and on eye-seeking *Musca sorbens* flies [3]. Promotion of face washing with soap to reduce person-to-person transmission of Ct has been a pillar of the WHO-endorsed SAFE strategy for trachoma control since the 1990s [4,5]. Although facial hygiene interventions have so far had limited impact on trachoma [6], absence of effect estimates of specific interventions may not mean that face washing with soap is ineffective in principle: as Ct bacteria are found in ocular and nasal discharge [7,8], both of which increase when a child has active trachoma [9], it is plausible that removing discharge from a child’s face reduces the cycle of transmission and reinfection that progresses the disease. Trachoma recrudescence and persistence in hyper-endemic areas suggest current strategies focussed on mass drug administration with azithromycin will not be sufficient to achieve long-term trachoma control in all regions [10–13]. In the context of global trachoma elimination, there is a recognised need for novel face washing programmes that can realise and sustain improvements in face washing with soap [14]. Accurate measurement of face washing behaviour will be vital to the monitoring, evaluation and strengthening of these programmes.

### Observation: The (imperfect) gold standard for measuring face washing behaviour

Accurately measuring behaviour to quantify the level of success of a behaviour change intervention is a common challenge in public health [15–17]. There is currently no single, easy-to-use, reliable indicator available to assess whether a face has been washed with soap. One approach is to measure face washing behaviour *in situ* through direct observation. Observation allows detailed, objective information to be collected on the actual behaviour of multiple individuals simultaneously. To do this an observer must sit unobtrusively in the location where the behaviour is anticipated to take place and record when a face is washed. This is possible when faces are washed outside, but more difficult in settings where face washing takes place in private or away from the home. Although often considered the ‘gold standard’ for measuring hygiene behaviours [18], observation is not without its drawbacks. Observation can be time-consuming and costly. It is also potentially subject to a particular form of social desirability bias known as the Hawthorne effect, whereby the individuals being observed deliberately or subconsciously alter their behaviour on account of the presence of the observer [19–21]. Furthermore, the nature of observation of face washing is fundamentally different from observation of other more commonly-studied hygiene behaviours such as hand washing, which is typically recorded as preceding or following another activity of interest, such as defecation. If face washing is not observed at a time when observation takes place, few instances of face washing will be recorded [22]. Face washing behaviour following an intervention has been directly measured through structured observation in schools [23], but behaviour has not been directly measured in any trials of face washing interventions [24,25].

### Alternative metrics for measuring face washing behaviour

A wide range of metrics have been developed to complement structured observation of handwashing behaviour [18,26–31], but fewer alternatives are in use to measure face washing. These include self-report [22,24,32–34], measurement of proxies for behaviour such as observation of soap and water availability [24,35] and visual assessment of various indicators of facial cleanliness [36–40]. The most recent trial of a face washing intervention assessed self-reported face washing behaviour and presence of wash facilities and soap [24], while another well-known trial of a face washing intervention reported facial cleanliness [25]. However, one study (published since the current trials were done) has assessed whether facial cleanliness assessed qualitatively or through qPHAT is a valid measure of face washing behaviour [41] but no studies to assess the validity of the other metrics have been performed. The limited available evidence on the validity of face washing metrics limits our ability to evaluate face washing interventions and in turn to learn how to develop more effective interventions.

These metrics have several limitations despite their potential utility to rapidly collect a large amount of data. Self-reported behaviour is associated with several forms of bias [42,43] and often results in substantial over-estimation of desirable practices [44,45], although this has not been studied for face washing. Proxy indicators such as soap availability can tell us (from its absence) if soap is *not* (currently) being used to perform a particular hygiene behaviour [26,29], but the reverse cannot be concluded if soap is present. ‘Clean face’ measures are popular because if a face is clean, it is plausibly less likely that trachoma will be transmitted–irrespective of whether measured associations between clean faces and absence of trachoma are real or are due to reverse causality or confounding [1,3,46]. However, the evidence supporting use of these metrics is mixed. A recent study found observation of oculo-nasal discharge could reliably detect recent face washing [41]. Other studies have suggested that qualitative, binary facial cleanliness metrics such as presence or absence of oculo-nasal discharge are not a good predictor of recent face washing [38,39]. The studies posit that this may be because dirt and discharge re-appear rapidly after washing (particularly if a child has trachoma and produces more discharge [47]), or could simply be because binary metrics are not sensitive enough to capture that a face wash has improved cleanliness, as a face will only be recorded as clean if it is completely devoid of the indicator in question. These metrics may have value in assessing trends over time, but more evidence is needed to determine whether qualitative facial cleanliness metrics are appropriate for assessing the success of face washing programmes [39]. The novel quantitative personal hygiene assessment tool (qPHAT) was developed to try to combat some of the limitations of the qualitative metric [40]. A recent study found negative associations between qPHAT scores and reported behaviour, suggesting the qPHAT metric may be less biased than reported behaviour [48]. One study suggests that the tool may have utility as a proxy for face washing behaviour [41].

This study aims to add to the body of evidence by comparing survey (self)-report, script-based pictorial recall and facial cleanliness metrics with structured observation for the measurement of face washing (with soap). The study findings will be used to inform behavioural outcome measures in the Stronger-SAFE trial [49].

## Methods

### Ethics and consenting

Ethical approval for the study was given within the approval granded for the Stronger-SAFE trial by the Ethics Boards at the London School of Hygiene & Tropical Medicine (Reference 17494), the Oromia Regional Health Bureau (BEFO/DDFDHU/1-89/3515), the National Research Ethics Review Committee of the Ethiopian Federal Ministry of Science and Technology (MOSHE//RD/141/8082/19) and the Ethiopian Food and Drug Authority (02/25/32/206). Written informed consent was obtained from all adult participants and parents/guardians of all children below the age of 18 years. Written assent was also taken from children aged 10–12.

Specific permission for data to be reported anonymously to communicate the findings of this research was sought from all adults and on behalf of all children under 18. It was made explicit that samples and information collected in this study could potentially be seen by researchers and students in the UK and beyond, and by health professionals and decision-makers in Ethiopia/UK and beyond.

### Study setting and population

This methods study was embedded in the baseline of the Stronger-SAFE cluster-randomised controlled trial between March and May 2021. Stronger-SAFE is a four-arm trial involving 68 clusters, designed to test whether enhanced azithromycin treatment (two doses, two weeks apart) combined with targeted transmission-interrupting strategies (face washing and fly control) can more effectively eliminate trachoma than current approaches [49]. A cluster contains approximately 90 households. The trial is being conducted around 250 km south of Addis Ababa in the rural West Arsi Zone in the Oromia region of Ethiopia. The face washing intervention aims to improve face washing with soap through provision of wash stations with soapy water accompanied by a series of events delivered at community, small-group and household level. All household members are targeted by the intervention, but particular emphasis is placed on the behaviour of pre-school age children (1–6 years old) due to their importance in trachoma transmission [25].

Data on face washing practices were collected from three households per cluster at baseline and three-month follow-up. The sample size was calculated to allow us to detect a pre-defined minimum difference in prevalence of three indicators of face washing with soap between study arms at the follow-up visit with 90% power. The baseline study that these data are drawn from used the same sample size. Eligible households had at least two children aged 1–12, of which at least one child was aged 1–6 years due to our interest in the behaviour of pre-school children. Invited households were selected randomly at each time point from a sampling frame based on a baseline census for the trial. A risk factor survey collecting basic socio-demographic data and information on water, sanitation and hygiene facilities was conducted during consenting on the day prior to data collection. Trachoma prevalence was assessed at baseline following procedures outlined in the Stronger-SAFE trial protocol [49].

### Face washing metrics

Structured observation was carried out for three hours from dawn in all households. This time period was chosen because it was not feasible to conduct day-long observations in the trial, and most face washing occurs at home in the early morning in this setting [50]. Enumerators were provided transport to help ensure they were in place by 6 am. Actual observation start and end times were recorded and verified by supervisors who accompanied the team to the field each day. Several measures were taken to minimise reactivity: (1) recruited enumerators were local to the study area but unconnected to the trial or other health promotion activities; (2) enumerators were trained to be discreet when recording data and non-judgmental in their verbal and non-verbal interactions with the family; (3) as we have done previously [51], participants were told that their daily activities and water use were being observed rather than observation of face washing explicitly; (4) a brief, unrelated survey on possession of household items such as toothpaste and coffee was administered during consenting on the day prior to observation to distract participants from the purpose of the observation. The observation tool consisted of a structured observation form that we had previously developed for other face washing studies in Ethiopia [50,52], and captured coded information about every body wash that took place during the observation period. A face wash was defined as any instance when the face was intentionally wet and rubbed (regardless of the perceived effectiveness of these actions). We recorded the timing, whose face was washed, who washed this person’s face, whether soap was used and whether the face was dried after washing. A short description of the activity was also recorded for quality control purposes.

Following observation, the primary caregiver in each household participated in a survey to capture self-reported behaviour and/or a routine-based script activity (described below) to covertly capture information on face washing behaviour, hereafter referred to as ‘pictorial recall’. These additional methods to measure actual face washing behaviour were employed to inform decisions about the set of measures we would use to evaluate the face washing intervention delivered in the trial. To avoid overburdening the respondents and minimise the risk of reporting bias, not all methods were applied in all households (Table 1): Group A households participated in pictorial recall; Group B households provided reported data; and Group C households participated in both methods. In Group C households the pictorial recall activity was conducted before the survey to minimise reporting bias. Primary caregivers provided information on their own behaviour and reported on the practices of all children aged 1–12 years. Children aged 4–12 years also participated in a simplified version of the pictorial recall and reported on their hygiene behaviour in select households. The facial cleanliness of children aged 1–12 years was assessed qualitatively and quantitatively once the survey and/or pictorial recall activity had been completed to minimise reporting bias.

**Table 1:** Face washing metrics collected at baseline.

Metric	Description	Indicator	Level	Sample Group	Sample Size
Structured observation	Continuous observation of all hygiene behaviours performed by household members for 3 hours in the early morning	Observed face washing with water (and soap) between 6am and 9am	Individual–all household members	A, B & C	877caregivers and children aged 1–12 years in 204 households
Script-based pictorial recall	Interview activity producing a script chronicling all tasks performed on the previous day. Subsequent enquiry includes specific prompts for activities involving water (and soap) use.	Unprompted (and prompted) recall of face washing with water (and soap) on previous morning from waking until midmorning (11am)	Individual–Caregiver (self-report)	A & C	134 caregivers in 134 households
	Interview activity producing a script chronicling water use activities performed on the previous day and any soap use during these activities. Specific prompts guide recall.	Prompted recall of face washing with water (and soap) on previous morning from waking until midmorning (11am)	Individual–Children aged 1–12 years (caregiver-report)	A & C	412 Children aged 1–12 years in 134 households
			Individual–Children aged 4–12 years (self-report)	A	142 Children aged 4–12 years in 68 households
Survey report	Structured survey asking whether the participant washed their face on the previous day and if so, how many times their face was washed with water, how many times with water and soap and when during the day these face washes took place.	Reported face washing with water (and soap) on previous morning after waking	Individual–Caregiver (self-report)	B & C	134 Caregivers in 134 households
			Individual–Children aged 1–12 years (caregiver-report)	B & C	413 Children aged 1–12 years in 136 households
			Individual–Children aged 4–12 years (self-report)	B	135 Children aged 4–12 years in 68 households
Qualitative facial cleanliness assessment	Assessment of presence or absence of ocular and nasal discharge and flies on children’s faces during 3-second observation in natural light while facing the child	Presence/absence of oculo-nasal discharge and flies on face	Individual–Children aged 1–12 years	A, B & C	619 Children aged 1–12 years in 204 households
Quantitative facial cleanliness assessment	Use of the quantitative personal hygiene assessment tool (qPHAT) (40), involving application of a face wipe to a child’s face and assessing its dirtiness.	Colour of the darkest area of a face wipe compared against an 11-point colour scale, wherein 0 represents the darkest colour and 10 the lightest.	Children aged 1–12 years	A, B & C	619 Children aged 1–12 years in 204 households

Data were collected in three randomly–selected households per cluster. Pictorial recall, survey report and facial cleanliness assessments took place at the end of the structured observation session to minimise bias. To avoid overburdening the respondents and minimise the risk of reporting bias, not all methods were applied in all households (Table 1): Group A households participated in pictorial recall; Group B households provided reported data; and Group C households participated in both methods, with pictorial recall data collected before the survey. Facial cleanliness assessments took place after all other data collection to minimise bias. One household per cluster was assigned to each group.

Script-based pictorial recall seeks to capture information on face washing practices without explicitly asking about them. A script is a spatially-temporally organised sequence of events [53]. Asking people to recount their daily activities in chronological order–a process known as ‘script elicitation’ in cognitive psychology [53]–can improve a person’s ability to recall their daily activities [54]. The ‘sticker diary’ covert recall method, developed by Unilever-Lifebuoy to measure hand washing behaviour [31], is based on script methodology. A respondent uses a series of stickers representing different daily activities to record their routine on the preceding day. We evaluated the sticker diary methodology and found diary estimates of hand washing with soap were comparable with structured observation data for some hand washing events, but over-reported others [31]. We adapted the ‘sticker diary’ methodology to our study context to see whether it could provide a valid alternative to structured observation to measure face washing behaviour.

We developed a set of daily routine cards using script elicitation techniques we have employed in other studies [52, 55] to ensure the cards were locally relevant. These cards depicted common daily activities (e.g. washing dishes, sweeping, cooking, etc.), along with personal hygiene behaviours of interest (face washing, hand washing, other body washing and a full bath). All personal hygiene behaviour cards had “with” and “without soap” options. Respondents were informed that we were interested in understanding daily water use and were asked to describe all the activities they undertook on the previous day. The day was broken into four sections to aid recall: morning; lunchtime; the afternoon; and the evening. Meal times were used to anchor the routine. Cards were laid down in front of the respondent to visually describe their routine as they mentioned each activity and they were encouraged to look back over their day and add in anything that had been missed. The final order of cards for this *unprompted* routine was recorded. Respondents were then reminded that the study was about water use and they were shown specific water use cards (including face washing) in turn. Images with and without soap were shown for each water use activity with neutral facial expression. Respondents were invited to select any applicable cards and insert them into their routine. The same activity could be inserted multiple times. The *prompted* daily routine was recorded when the respondent was satisfied it was complete. The respondent completed a daily routine for all children aged 1–12 in the household. As the caregiver had already been prompted about face washing, asking them to recount unprompted routines for their children was susceptible to bias, so we only collected prompted routines for children. If the tool showed promise, it also needed to be feasible to collect data on multiple children in a household, so we developed a simplified, prompted pictorial recall activity focused on water use activities performed with or without soap (washing dishes, washing clothes, hand washing, face washing, other body washing and a full bath). As we were interested to learn whether maternal or self-report is more accurate, children aged 4–12 were also invited to participate in the modified *prompted* pictorial recall activity. School and meal cards were used as relevant to help segment the day and improve recall. Final routines were copied from paper forms to Open Data Kit (ODK) forms, an electronic mobile data collection platform.

Self-reported behaviour was captured through a face-to-face ODK survey with verbally posed questions:

- Did you wash your face at all yesterday?
- How many times did you wash your face with water only yesterday?
- How many times did you wash your face with water and soap yesterday?

These questions were piloted with various phrasings to reduce social desirability bias before the questions were finalised. The same questions were used to capture caregiver reported behaviour for 1–12 years olds in the household and to question 4–12 year olds directly on their own behaviour. Participants were also asked when they washed their faces with water and soap on the previous day in relation to specific times of day. At the end of the survey the HWISE scale was used to enquire about household water insecurity [56].

Facial cleanliness was assessed after completion of the survey by observing the front of each child’s face, outside in natural daylight for three seconds. We recorded presence of ocular discharge, nasal discharge and flies on the face. A face was considered clean in the absence of oculo-nasal discharge following King et al. [38]. Facial cleanliness was subsequently assessed using the quantitative personal hygiene assessment tool (qPHAT) [40]. Each child’s eye was wiped with a moist wipe in a systematic way following Stronger-SAFE trial protocols and the qPHAT methodology. Using gentle pressure, the wipe traced the skin from the ear, across the top of the eyelid, down along the tear duct and back to the ear following the skin under the eye. The colour of the darkest area of the wipe was compared against an 11-point colour scale, wherein 0 represents the darkest colour and 10 the lightest.

### Follow-up data collection

A new random sample of 204 households (3 per cluster) was selected for interim behavioural outcome assessment in 2022, three months after delivery of the main face washing intervention in the Stronger-SAFE trial. Following review of baseline data, outcomes were assessed through structured observation and script-based pictorial recall, but not through survey (self)-report. Following experiences using the tool at baseline, the script-based pictorial recall exercise was simplified to only capture activities related to water use, and self-report by children was limited to children aged 7–12 years-of-age.

### Training

Enumerators were trained on the study tools and research principles in the classroom and the field over 10 days. Grading of facial cleanliness indicators was standardised during training through comparison with “master” rater grades assigned by an experienced trainer with repeat training as required. All enumerators who participated in the study achieved chance-corrected agreement on qualitative indicators (Cohen’s kappa statistic) of > = 0.90. Following de Lea et al. [40], inter-rater reliability of qPHAT scores was assessed using Gwet’s agreement coefficient and quadratic weights. Enumerators were deemed competent when coefficients were > = 0.90 *and* enumerator grades were within 1-point of the master rater.

### Statistical methods

As every observed face washing event was linked to an individual on the household census, we were able to document the face washing status of all household members present during the observation period, so we knew who washed with water, who washed with soap and who did not wash at all. Binary variables were created to describe whether an individual washed their face during observation (regardless of soap use), and to identify those who washed their faces with soap. If a face was washed more than once during the observation period, it was defined as having been washed with soap if soap was used on any face wash occasion. Similar variables were created to describe an individual’s face wash status in the pictorial recall and reported behaviour datasets. All instances where a “full bath” was described in the pictorial recall were defined as a face wash. Separate variables were created for prompted and unprompted accounts of face washing during pictorial recall. Nine records of caregiver self-report, parental report and child self-report with “don’t know” recorded in response to the question about whether a face was washed on the previous day were excluded from further analysis. A further 80 of 692 reports responded “don’t know” in response to questions about use of soap during face washing. Data were analysed separately for caregivers, pre-school children 1–3 years old, pre-school children 4–6 year olds and primary school age children 7–12 years old.

Differences between distribution of categories of WASH characteristics across the three groups were assessed using Pearson’s chi-squared test with Fisher’s Exact Test applied when sample size was small. Metric validity was assessed in several ways. Mean cluster-level prevalence estimates and associated 95% confidence intervals were computed and compared using t tests. Prevalence differences between face washing and face washing with soap estimates for each indicator and observed behaviour were obtained from binomial regression analysis (binomial distribution, identity link) which accounted for the paired nature of the data and adjusted for clustering using Generalised Estimating Equations. Analysis of survey data was conducted separately for Group B (survey only) and Group C (survey following pictorial recall) to assess for bias. Sensitivity analysis was performed on the group who were not observed to wash their faces to see whether pictorial recall findings differed by observation start time as an indication of the extent of face washing before the observation period commenced. The sensitivity, specificity, positive predictive value and negative predictive value of each indicator were computed and compared using structured observation as the gold standard. Pictorial recall data from baseline and interim follow-up were analysed in a restricted dataset that only contained data from individuals who had not been observed to wash their face (with or without soap) during the three-hour morning observation to assess for differential reporting bias in pictorial recall responses introduced as a result of exposure to the intervention. Cluster-level prevalence of face washing (with soap) and associated 95% confidence intervals were computed and means were compared between study arms at baseline and follow-up using t tests. Qualitative and quantitative facial cleanliness metrics were analysed for each age group and according to the time lag between observed face washing and facial cleanliness assessment using Pearson’s chi-squared or Fisher’s exact tests (clean face comparisons) or t tests and Mann-Whitney tests (face wipe scores).

## Results

### Characteristics of study population

Prevalence of the active trachoma sign “trachomatous inflammation—follicular (TF) in 1-9-year-olds in trial clusters at baseline was 30%. Socio-demographic characteristics of the 204 participating households were broadly similar in each group (Table 2). All but three households practised Islam and two-thirds (66% of 204 respondents) self-assessed that they had ‘average’ wealth compared with other households in their village. The median age of female primary caregivers participating in the survey or pictorial recall activity was 30 years (range 18 to 60); 70% of these respondents had not received any formal education. Table 3 shows the WASH context in participating households at baseline. Three-quarters of respondents had only basic water access, reporting that it took over 30 minutes to collect water from a public tap or standpipe. Seventeen percent of households had no water anywhere on their plot at the time of the survey and 85% of households were determined to be ‘water insecure’ according to the HWISE scale [56]. Forty-one percent of households had access to a latrine, of which most were simple pit latrines with no slab. Thirty-eight percent of households had soap at the time of the survey, but only 13% of households reported that they had not had soap on any day in the previous week. The WASH context was comparable across groups (Table 3).

**Table 2:** Socio–demographic characteristics of baseline study households (N = 204).

	Group A Households (N = 68)	Group B Households (N = 68)	Group C Households (N = 68)	Total (N = 204)
Household size, mean (SD)	6.7 (2.1)	6.6 (2.1)	6.0 (1.9)	6.4 (2.0)
Religion				
Muslim	65 (98.5%)	66 (98.5%)	67 (98.5%)	198 (98.5%)
Christian	1 (1.5%)	1 (1.5%)	1 (1.5%)	3 (0.5%)
Female primary caregiver, median age (range)	30 (18–60)	30 (20–50)	30 (19–50)	30 (18–60)
Caregiver formal education, mean years (SD)	1.8 (3.0)	1.3 (2.4)	1.2 (2.0)	1.4 (2.5)
Proportion of primary caregivers with no formal education, n (%)	45 (68.2%)	49 (72.1%)	45 (67.2%)	142 (69.6%)
Number of rooms, mean (SD)	1.6 (0.61)	1.51 (0.61)	1.6 (0.53)	2.1 (0.75)
Household assets, n (%)				
Land for farming	65 (98.5%)	65 (95.6%)	67 (100%)	200 (98.0%)
Donkey car	20 (30.3%)	23 (33.8%)	20 (29.9%)	65 (31.9%)
Electricity	6 (9.1%)	5 (7.4%)	4 (6.0%)	15 (7.4%)
Solar lamp or power	10 (15.2%)	19 (27.9%)	13 (19.4%)	42 (20.6%)
Working mobile telephone	40 (60.6%)	37 (54.4%)	41 (61.2%)	119 (58.3%)
Working television	0	0	0	0
Self-assessed wealth, n (%)				
Very poor	4 (6.1%)	4 (5.9%)	5 (7.5%)	13 (6.4%)
Poor	19 (28.8%)	16 (23.5%)	15 (22.4%)	51 (25.0%)
Average	41 (62.1%)	47 (69.1%)	45 (67.2%)	135 (66.2%)
Wealthy	2 (3.0%)	1 (1.5%)	2 (3.0%)	5 (2.5%)

Denominators vary due to occasional missing data.

**Table 3:** WASH context in baseline study households (N = 204).

	Group A Households (N = 68)	Group B Households (N = 68)	Group C Households (N = 68)	Total (N = 204)	*P-*value
Main drinking water source according to JMP Service Ladder*, n (%)					
Surface water	0	1 (1.5%)	2 (3.0%)	3 (1.5%)	0.963
Unimproved service (unprotected spring or well)	1 (1.5%)	1 (1.5%)	1 (1.5%)	3 (1.5%)	
Limited service (improved source, > 30 min round trip)	53 (80.3%)	51 (75.0%)	49 (73.1%)	153 (75.0%)	
Basic service (improved source, < 30 min round trip)	6 (9.1%)	6 (8.8%)	6 (9.0%)	19 (9.3%)	
Improved source on premises	6 (9.1%)	9 (13.2%)	9 (13.4%)	26 (12.8%)	
Proportion of households with no stored water on plot at time of survey, n (%)	15 (22.7%)	16 (23.5%)	13 (19.1%)	31 (17.2%)	0.825
Proportion of households determined to be water insecure according to HWISE Scale score^, n (%)	59 (89.4%)	56 (82.4%)	57 (83.8%)	172 (85.2%)	0.483
Latrine access, n (%)	27 (40.9%)	24 (35.3%)	32 (47.8%)	84 (41.2%)	0.338
Latrine type, n (%)					
Open pit, or pit latrine with no slab	22 (81.5%)	21 (87.5%)	25 (78.1%)	69 (82.1%)	0.951
Pit latrine with slab	4 (14.8%)	3 (12.5%)	6 (18.8%)	13 (15.5%)	
VIP (ventilated improved pit) latrine	1 (3.7%)	0	1 (3.1%)	2 (2.4%)	
Observed soap available in home, n (%)	28 (42.4%)	29 (42.7%)	20 (29.9%)	78 (38.2%)	0.218
Reported availability of soap–last 7 days					
Every day	4 (6.1%)	9 (13.2%)	11 (16.18)	24 (11.9%)	0.100
Most days	23 (34.9%)	10 (14.7%)	19 (27.9%)	52 (25.7%)	
Some days	30 (45.5%)	39 (57.4%)	30 (44.1%)	99 (49.0%)	
No days	9 (13.6%)	10 (14.7%)	8 (11.8%)	27 (13.4%)	

Denominators vary due to occasional missing data.

* WHO/UNICEF Joint Monitoring Program (JMP) service ladder [57]. Variable created from data on the main source of drinking water and the round–trip time taken to collect water, including waiting time. The ‘Improved source on premises’ category is included in place of ‘Safely managed’, as it is unknown whether the source is available when needed and free of contamination.

^ Variable created from 12 variables used to assess different components of water scarcity [56].

### Face washing prevalence estimates by metric

Early morning face washing habits of 877 caregivers and children aged 1–12 in 204 households in 68 clusters were captured through structured observation. Seventy-one percent of observation sessions had commenced by 06:15 and 82% by 06:30 (range 05:47 to 07:09). Face washing was observed in 58 of the 145 households where observation had commenced by 06:15. Seventy-eight percent of 344 faces washes in these households took place after 06:30.

Face washing was observed to be performed by 39% of 199 caregivers (4% with soap), 61% of 404 1–6-year-olds (5% with soap) and 51% of 274 7–12-year-olds (2% with soap). Eighty-eight percent of 106 face washes among 1–3-year-olds and 21% of 138 face washes among 4–6-year-olds were performed by a caregiver. All but two of the 139 face washes observed among school-age children 7–12-year-olds were self-washed. Seventy-four percent of faces were not dried after washing, 17% were dried using a hand or arm, 9% with clothing and 1% with a rag or towel.

Table 4 compares the observed prevalence of face washing and face washing with soap with prevalence estimates obtained through pictorial recall and respondent report. According to survey (self)-report, almost all caregivers and children washed their faces shortly after waking on the previous morning, resulting in prevalence estimates 32% to 60% higher than the observed data. Estimates obtained through child self-report closely matched behaviour reported by caregivers for the same children: 94% of 4–6-year-olds and 98% of their caregivers reported face washing on the previous day (*P* = 0.42), while 95% of 7–12-year-olds and 97% of their caregivers reported face washing on the previous day (*P* = 0.60). Survey estimates of face washing with soap were also higher than observed behaviour, except for self-reported estimates provided by the youngest children in the study (Table 4). These children reported substantially less face washing with soap than their caregivers reported for them (9% vs 27%, *P* = 0.01). Conducting the survey after pictorial recall did not significantly alter face washing prevalence estimates.

**Table 4:** Comparison of face washing (with soap) prevalence estimates from structured observation with pictorial recall and survey (self–) report.

		Any Face washing			Face washing with soap		
Metric	Indicator	N	Prevalence (95% CI)	Prevalence Difference (95% CI)	N	Prevalence (95% CI)	Prevalence Difference (95% CI)
**CAREGIVERS**							
Structured observation	Observed face washing between 6am and 9am	199	38.6% (30.9%–46.3%)	Ref.	199	4.4% (1.3%–7.5%)	Ref.
Survey–Caregiver self-report	Self-reported face washing on previous morning after waking	134	98.5% (96.5%–100%)	+60.0%** (54.0%–65.9%)	123	36.0% (27.0%–45.1%)	+31.2%** (22.2%–40.2%)
Script-based Pictorial recall–Caregiver self-recall	Unprompted recall of face washing on previous morning from waking until midmorning (11am)	134	21.8% (13.9%–29.7%)	-15.8%* (-25.9%–-5.7%)	134	7.1% (2.4%–11.8%)	+3.0% (-2.4%–8.4%)
	Prompted recall of face washing on previous morning from waking until midmorning (11am)	134	78.7% (71.3%–86.0%)	+40.6%** (30.8%–50.5%)	134	15.9% (9.6%–22.3%)	+12.6%** (5.7%–19.6%)
**PRE-SCHOOL CHILDREN 1–3 YEARS**							
Structured observation	Observed face washing between 6am and 9am	180	61.6% (53.9%–69.3%)	Ref.	180	4.5% (0.9%–8.1%)	Ref.
Survey–Caregiver report	Caregiver-reported face washing on previous morning after waking	112	96.7% (93.4%–100%)	+36.6%** (27.7%–45.6%)	103	32.1% (20.9%–43.4%)	+27.9%** (18.6%–37.2%)
Script-based Pictorial recall–Caregiver recall	Prompted caregiver recall of face washing on previous morning from waking until midmorning (11am)	112	84.3% (76.0%–92.5%)	+24.3%** (14.5%–34.2%)	112	15.4% (7.4%–23.4%)	+12.6%* (5.1%–20.2%)
**PRE-SCHOOL CHILDREN 4–6 YEARS**							
Structured observation	Observed face washing between 6am and 9am (children 4–6 years old only)	224	63.5% (56.7%–70.3%)	Ref.	224	4.4% (0.6%–8.1%)	Ref.
Survey–Caregiver report	Caregiver-reported face washing on previous morning after waking (children 4–6 years old only)	143	97.7% (95.0%–100%)	+35.1%** (27.7%–42.5%)	126	27.3% (17.9%–36.7%)	+23.3%** (15.5%–31.2%)
Survey–Child self-report	Self-reported face washing on previous morning after waking (children 4–6 years old only)	54	94.2% (87.3%–100%)	+31.8%** (22.9%–40.7%)	49	8.5% (0%–17.1%)	+5.0% (-2.8%–12.8%)
Script-based Pictorial recall–Caregiver recall	Prompted caregiver recall of face washing on previous morning from waking until midmorning (11am) (children 4–6 years old only)	140	86.2% (79.3%–93.1%)	+25.3%** (17.0%–33.7%)	140	8.2% (2.7%–13.7%)	+5.8%* (0.5%–11.1%)
Script-based Pictorial recall–Child self-recall	Prompted recall of face washing on previous morning from waking until midmorning (11am) (children 4–6 years old only)	61	71.6% (58.3%–84.9%)	+14.3%* (2.0%–26.7%)	61	4.5% (0%–100%)	+3.4% (-3.2%–10.1%)
**SCHOOL-AGE CHILDREN 7–12 YEARS**							
Structured observation	Observed face washing between 6am and 9am	274	50.6% (43.0%–58.2%)	Ref.	274	2.0% (0%–4.3%)	Ref.
Survey–Caregiver report	Caregiver-reported face washing on previous morning after waking	158	97.3% (93.7%–100%)	+43.8%** (36.7%–51.0%)	138	18.1% (9.8%–26.4%)	+19.6%** (12.8%–26.4%)
Survey–Child self-report	Self-reported face washing on previous morning after waking	81	95.1% (89.3%–100%)	+42.4%** (34.4%–50.4%)	70	17.8% (8.0%–27.6%)	+16.0%* (6.9%–25.1%)
Script-based recall–Caregiver recall	Prompted caregiver recall of face washing on previous morning from waking until midmorning (11am)	160	81.9% (74.4%–89.4%)	+27.7%** (19.2%–36.3%)	160	1.2% (0%–2.7%)	+0.3% (-2.3%–2.9%)
Script-based recall–Child self-recall	Prompted recall of face washing on previous morning from waking until midmorning (11am)	81	71.9% (59.9%–83.9%)	+16.7%* (5.1%–28.4)	81	5.2% (0%–11.4%)	3.4% (-0.2%–8.4%)

As not all households and individuals participated in all methods, denominators differ for each indicator. To minimise bias, the survey asked how many times faces were washed with soap as opposed to directly asking whether a face was washed with soap and consequently there was a small amount of missing data. Proportions shown are cluster–level mean proportions. Prevalence differences account for whether data are paired and are marked with * *P*< = 0.05 and ** *P*< = 0.001 to indicate the level of significance of differences between tested metrics and observed data.

Face washing prevalence estimates from pictorial recall were lower than survey report, but almost always higher than observed behaviour, particularly when caregivers answered on behalf of their children (86% vs. 72% self-report by children aged 4–6 years, *P* = 0.08; 82% vs. 72% self-report by children aged 7–12 years; *P* = 0.04). Caregiver unprompted and prompted pictorial recall produced very different results: unprompted recall = 22% prevalence (16% lower than observed behaviour), prompted recall = 79% prevalence (41% higher than observed behaviour) (*P*<0.001) (Table 4). In contrast, face washing with soap prevalence estimates from pictorial recall were comparable with structured observation data (Table 4): there were no statistically significant differences between observed data and unprompted caregiver pictorial recall (3% prevalence difference, *P* = 0.28); pictorial recall by children 4–6 years old (self-recall 3% prevalence difference, *P* = 0.31); or pictorial recall by children aged 7–12 years (0% prevalence difference, *P* = 0.18) or their caregivers (3%, *P* = 0.83). Sensitivity analysis of caregivers who were not observed to wash their faces found stratifying by observation start time did not change pictorial recall findings, indicating differences between pictorial recall and observed data are more likely due to reporting rather than missed observations.

### Sensitivity, specificity and predictive values of face washing metrics

Table 5 shows the sensitivity, specificity and predictive values for each metric using structured observation as the gold standard. Pictorial recall indicators for face washing with soap have a reasonably high specificity (ranging from 85% to 99% across the metrics), but low sensitivity (0% to 67%). However, sensitivity estimates are very imprecise due to the low number of participants who were observed washing their face with soap, resulting in a low positive predictive value. This suggests that while the pictorial recall indicators for face washing with soap gave prevalence estimates that were reasonably close to the estimates from the gold standard, this was just by chance as most of the participants who reported face washing with soap through pictorial recall were false positives. As pictorial recall missed almost everyone who actually washed their face, in a higher prevalence setting we could expect pictorial recall to underestimate the prevalence of face washing with soap.

**Table 5:** Sensitivity, specificity, positive predictive value and negative predictive value of face washing (with soap) metrics compared to structured observation.

		Any Face washing				Face washing with soap			
Metric	Indicator	Sensitivity (95% CI)	Specificity (95% CI)	Positive Predictive Value (95% CI)	Negative Predictive Value (95% CI)	Sensitivity (95% CI)	Specificity (95% CI)	Positive Predictive Value (95% CI)	Negative Predictive Value (95% CI)
**CAREGIVERS**									
Survey–Caregiver self-report	Self-reported face washing on previous morning around time of waking	100% (92.1%–100%)	2.4% (0.3%–8.3%)	35.4% (27.2%–44.4%)	100% (15.8%–100%)	50% (6.8%–93.2%)	65.2% (55.8%–73.9%)	4.8% (0.6%–16.2%)	97.4% (90.9%–99.7%)
Script-based Pictorial recall–Caregiver self-recall	Unprompted recall of face washing on previous morning from waking until midmorning (11am)	30.8% (18.7%–45.1%)	82.9% (72.5%–90.6%)	55.2% (35.7% -73.6%)	63.6% (53.4%–73.1%)	20.0% (0.5%–71.6%)	93.5% (87.6%–97.2%)	11.1% (0.3%–48.2%)	96.6% (91.6%–99.1%)
	Prompted recall of face washing on previous morning from waking until midmorning (11am)	76.9% (63.2%–87.5%)	19.7% (11.5%–30.5%)	39.6% (30.0%–49.8%)	55.6% (35.3%–74.5%)	60.0% (14.7%–94.7%)	85.4% (77.9%–91.1%)	14.3% (3.1%–36.3%)	98.1% (93.4%–99.8%)
**PRE-SCHOOL AGE CHILDREN 1–3 YEARS**									
Survey–Caregiver report	Caregiver-reported face washing on previous morning around time of waking	92.5% (83.4%–97.5%)	0% (0%–7.9%)	57.9% (48.0%–67.4%)	0% (0%–52.2%)	50% (6.8%–93.2%)	69.7% (59.6%–78.5%)	6.3% (0.8%–20.8%)	97.2% (90.2%–99.7%)
Script-based Pictorial recall–Caregiver recall	Prompted caregiver recall of face washing on previous morning from waking until midmorning (11am)	88.7% (79.0%–95.0%)	24.4% (12.4%–40.3%)	67.0% (56.6%–76.4%)	55.6% (30.8%–78.5%)	40.0% (5.3%–85.3%)	85.0% (76.9%–91.2%)	11.1% (1.4%–34.7%)	96.8% (91.0%–99.3%)
**PRE-SCHOOL AGE CHILDREN 4–6 YEARS**									
Survey–Caregiver report	Caregiver-reported face washing on previous morning around time of waking (children 4–6 years old only)	96.6% (90.4%–99.3%)	1.8% (0%–9.7%)	61.2% (52.5%–69.3%)	25.0% (0.6%–80.6%)	0% (0%–84.2%)	73.4% (64.7%–80.9%)	0% (0%–10.6%)	97.6% (92.4%–99.7%)
Survey–Child self-report	Self-reported face washing on previous morning around time of waking (children 4–6 years old only)	94.7% (82.3%–99.4%)	6.3% (0.2%–30.2%)	70.6% (56.2%–82.5%)	33.3% (0.8%–90.6%)	0% (0%–97.5%)	92.0% (80.8%–97.8%)	0% (0%–60.2%)	97.9% (88.7%–99.9%)
Script-based Pictorial recall–Caregiver recall	Prompted caregiver recall of face washing on previous morning from waking until midmorning (11am) (children 4–6 years old only)	88.5% (79.9%–94.3%)	13.2% (5.5%–25.3%)	62.6% (53.4%–71.2%)	41.2% (18.4%–67.1%)	20.0% (0.5%–71.6%)	91.9% (85.9% (95.9%)	8.3% (21.1%–38.5%)	96.9% (92.2%–99.1%)
Script-based Pictorial recall–Child self-recall	Prompted recall of face washing on previous morning from waking until midmorning (11am) (children 4–6 years old only)	75.0% (58.8%–87.3%)	19.0% (5.5%–41.9%)	63.8% (48.5%–77.3%)	28.6% (8.4%–58.1%)	0% (0%–70.8%)	93.1% (83.3%–98.1%)	0% (0%–60.2%)	94.7% (85.4%–98.9%)
**SCHOOL-AGE CHILDREN 7–12 YEARS**									
Survey–Caregiver report	Caregiver-reported face washing on previous morning around time of waking	98.9% (94.0%–100%)	4.6% (1.0%–12.9%)	59.2% (51.0%–67.1%)	75.0% (19.4%–99.4%)	0% (0%–97.5%)	79.3% (71.4%–85.8%)	0% (0%–12.3%)	99.1% (94.9%–100%)
Survey–Child self-report	Self-reported face washing on previous morning around time of waking	95.3% (84.2%–99.4%)	3.0% (0.1%–15.8%)	56.2% (44.1%–67.8%)	33.3% (84.0%–90.6%)	100% (2.5%–100%)	83.6% (72.5%–91.5%)	8.3% (0.2%–38.5%)	100% (93.6%–100%)
Script-based recall–Caregiver recall	Prompted caregiver recall of face washing on previous morning from waking until midmorning (11am)	83.3% (74.0%–90.4%)	20.0% (11.4%–31.3%)	57.3% (48.3%–65.9%)	48.3% (29.4%–67.5%)	66.7% (9.4%–99.2%)	99.4% (96.5%–100%)	66.7% (9.4%–99.2%)	99.4% (96.5%–100%)
Script-based recall–Child self-recall	Prompted recall of face washing on previous morning from waking until midmorning (11am)	68.3% (51.9%–81.9%)	27.5% (14.6%–43.9%)	49.1% (35.6%–62.7%)	45.8% (25.6%–67.2%)	33.3% (0.8%–90.6%)	96.2% (89.2%–99.2%)	25.0% (0.6%–80.6%)	97.4% (90.9%–99.7%)

### Facial cleanliness metrics

The proportion of children aged 1–12 years with oculo-nasal discharge, flies on their face and a clean face (absence of oculo-nasal discharge) is shown in Table 6 stratified by recent observed face washing behaviour (the child did not wash, the child’s face was washed with water, or the child’s face was washed with water and soap). The mean time between observed face washing during the structured observation session and inspection of facial cleanliness was 2 hours 48 minutes (range: 8 minutes to 5 hours 37 minutes). Overall, 59% of children were seen to have their face washed during the observation period, 3% of whom washed with soap. Contrary to expectation, 17% of children who did not wash their faces had a clean face, compared with 8% of those who washed their face with water and 5% of those who washed with soap (Table 6). Facial cleanliness improved with increasing age: *P* = 0.03 for 4–6 year olds and *P*<0.001 for 7–12 year olds compared to the youngest age group. However, “non-washers” in the oldest age group had significantly cleaner faces than those who had washed with water (*P =* 0.003). Washing with soap may remove more ocular discharge, but the sample size is small.

**Table 6:** Ability of facial cleanliness to predict recent face washing (with soap) among children 1–12 years.

		Qualitative Facial Cleanliness Metrics					Quantitative Facial Cleanliness Metric				
Observed Face washing between 6am and 9am	N	Ocular discharge (%)	Nasal discharge (%)	Flies on face^#^ (%)	Prevalence of a Clean Face*	*P-*value for Clean Face	Mean qPHAT Score^	Median qPHAT Score^	95% CI	Range	*P-*value
**PRE-SCHOOL AGE CHILDREN 1–3 YEARS**											
None	69	81.2%	89.9%	53.6%	2.9%	Ref.	5.00	5	4.57–5.42	1 to 9	Ref.
Face washing with water	96	80.2%	94.8%	55.2%	1.0%	0.361	5.07	5	4.71–5.44	0 to 9	0.626
Face washing with soap	8	75.0%	75.0%	25.0%	1.7%	1.0	5.25	5	3.53–6.97	2 to 8	0.647
**PRE-SCHOOL AGE CHILDREN 4–6 YEARS**											
None	81	71.6%	82.7%	42.0%	11.1%	Ref.	4.65	5	4.27–5.04	1 to 9	Ref.
Face washing with water	126	75.4%	85.7%	42.9%	7.9%	0.445	4.85	5	4.59–5.11	1 to 9	0.478
Face washing with soap	7	85.7%	85.7%	57.1%	0	1.0	4.71	4	3.23–6.20	3 to 8	0.900
**SCHOOL AGE CHILDREN 7–12 YEARS**											
None	101	60.4%	51.5%	24.8%	30.7%	Ref.	5.14	5	4.78–5.50	2 to 9	Ref.
Face washing with water	120	68.3%	69.2%	35.8%	14.2%	0.003	5.06	5	4.73–5.39	1 to 9	0.802
Face washing with soap	4	25.0%	75.0%	0	25.0%	1.0	3.80	3.5	2.23–5.27	3 to 5	0.118

^#^ Assessed in daylight during 3 second observation.

* Clean face defined as absence of oculo–nasal discharge. Clean face assessed following the end of the structured observation period.

^qPHAT = quantitative personal hygiene assessment tool. Scores on an 11–point scale from 0 to 10, where 0 is the dirtiest and 10 is the cleanest [40]. Application of qPHAT immediately followed the clean face assessment at the end of the structured observation period.

Quantitative data on facial cleanliness did not differ significantly by face washing status or age group (Table 6). qPHAT scores did not vary by time since (last) face wash, even when analysis was restricted to face washes within 1.5 hours of assessment (N = 31), (0.06% of variation in qPHAT scores explained by time in minutes [*F*(1,29) = 0.18, *P* = 0.670].

### Use of pictorial recall to measure intervention effects

Table 7 shows face washing and face washing with soap estimates for caregivers, pre-school children and school age children obtained through pictorial recall at baseline and three months after delivery of a face washing intervention for an analysis restricted to individuals who were not observed to wash their face during a preceding structured observation session. As no individuals in this dataset washed their faces, the data indicate the extent of overreporting of face washing during pictorial recall in each study arm before and after intervention delivery. *P-*values for comparisons between study arms show there were no statistically significant differences in the level of reporting of face washing by caregivers or children between study arms at baseline, but several differences presented at follow up. Overall prevalence of caregiver face washing captured through pictorial recall was higher at follow-up than at baseline due to use of the simplified methodology that shortened the time taken to do the exercise. With exception of self-report by children 7–12 years-of-age, face washing with soap was significantly more frequently reported by individuals in the intervention arm who did not face wash (16% to 31%) than by those in the control arm who did not face wash (6% to 18%).

**Table 7:** Face washing (with soap) prevalence from script–based pictorial recall among individuals not observed to wash faces at baseline and follow–up, by study arm.

		Baseline					Follow-Up (3 months post-intervention)				
Metric	Indicator	N	Intervention (95% CI)	N	Control (95% CI)	*P*-value	N	Intervention (95% CI)	N	Control (95% CI)	*P*-value
***ANY FACE WASHING***											
***Caregivers***											
Script-based recall–Caregiver self-report	Unprompted recall of face washing on previous morning from waking until midmorning (11am)	38	18.5% (3.9%–33.2%)	38	14.8% (2.8%–26.9%)	0.70	65	74.0% (63.6%–84.4%)	70	60.4% (48.5%–72.3%)	0.08
	Prompted recall of face washing on previous morning from waking until midmorning (11am)	38	79.6% (64.8%–94.4%)	38	77.8% (62.9%–92.6%)	0.857	65	97.5% (94.0%–100%)	70	96.9% (93.3%–100%)	0.786
***Pre-School Children 1–6 years***											
Script-based recall–Caregiver report	Prompted caregiver recall of face washing on previous morning from waking until midmorning (11am)	51	87.2% (75.3%–99.1%)	40	82.6% (69.4%–95.8%)	0.598	134	89.3% (82.1%–96.5%)	129	87.3% (78.4%–96.2%)	0.723
***School age Children 7–12 years***											
Script-based recall–Caregiver report	Prompted caregiver recall of face washing on previous morning from waking until midmorning (11am)	32	83.3% (67.8%–98.9%)	37	72.0% (53.2%–90.7%)	0.361	90	80.5% (75.9%–90.1%)	98	85.4% (76.4%–94.4%)	0.498
Script-based recall–Child self-report	Prompted recall of face washing on previous morning from waking until midmorning (11am)	19	65.4% (36.8%–94.0%)	21	68.8% (43.2%–94.3%)	0.851	78	94.6% (87.3%–100%)	90	75.4%–96.1%)	0.164
***FACE WASHING WITH SOAP***											
***Caregivers***											
Script-based recall–Caregiver self-report	Unprompted recall of face washing on previous morning from waking until midmorning (11am)	62	5.9% (-1.3%–13.0%)	61	8.3% (0.6%–16.1%)	0.638	92	16.2% (9.5%–22.8)	97	6.4% (1.4%–11.3)	0.02
	Prompted recall of face washing on previous morning from waking until midmorning (11am)	62	11.8% (3.1%–20.4%)	61	17.2% (7.8%–26.5%)	0.392	92	29.9% (19.3%–40.5%)	97	18.1% (8.8%–27.5%)	0.09
***Pre-School Children 1–6 years***											
Script-based recall–Caregiver report	Prompted caregiver recall of face washing on previous morning from waking until midmorning (11am)	124	11.4% (4.1%–18.8%)	113	8.1% (1.8%–14.4%)	0.492	188	30.7% (20.1%–41.2%)	196	16.2% (7.5%–24.9%)	0.03
***School age Children 7–12 years***											
Script-based recall–Caregiver report	Prompted caregiver recall of face washing on previous morning from waking until midmorning (11am)	75	0 (0%–4.7%)	80	0.8% (0%–2.5%)	-	139	20.1% (10.2%–30.0%)	154	6.6% (1.2%–12.1%)	0.02
Script-based recall–Child self-report	Prompted recall of face washing on previous morning from waking until midmorning (11am)	38	4.3% (-4.7%–13.4%)	40	2.2% (-2.3%–6.7%)	0.657	123	19.4% (8.9%–29.8%)	145	13.0% (5.5%–20.5%)	0.31

Participants came from different random samples at baseline and follow–up. The pictorial recall exercise for caregivers was simplified at follow–up so it only captured daily activities related to water use (as opposed to the full daily routine). Child routines were captured in the same way at each time point.

## Discussion

This study assessed whether survey report, script-based pictorial recall and facial cleanliness indicators represent valid alternatives to costly, time-consuming structured observation for the measurement of face washing behaviour in community settings. None of the tested metrics appeared to accurately measure behaviour in this study. Alternative, easier to employ metrics cannot be recommended for the measurement of face washing behaviour.

Survey (self)-report overestimated both face washing and face washing with soap. Overinflated estimates of behaviour are commonly seen when a survey is used to estimate a normative behaviour [58–61], but this phenomenon has not previously been documented for face washing. Our study found that reported behaviour is not a good measure of actual practice. Conducting the pictorial recall after the survey did not significantly change the results, suggesting the methodology did not bias survey responses. Survey report is not being used to assess behaviour change outcomes in the Stronger-SAFE trial. Survey data may be useful to assess other outcomes, such as to identify where to target an intervention or to follow trends in soap use. Caution should be applied before collecting reported data to measure actual behaviour, particularly in the context of face washing with soap promotion: differential measurement bias between treatment groups may occur as a result of overreporting following intervention exposure [62, 63], making it difficult to corroborate measured intervention effects based on reported data.

The other metric tested to assess face washing behaviour was script-based pictorial recall, a novel application of this methodology. Even though they both collect retrospective data on behaviour, chronological, diary-based methodologies like pictorial recall have been shown to give less biased estimates of normative behaviours than surveys [64]. This is possibly because they avoid identity-related measurement biases associated with explicit questioning on a particular behaviour [61]. Use of script-based pictorial recall in our study produced estimates of face washing with soap that were comparable with structured observation.

At first glance, the metric shows promise as a complement or replacement for structured observation. However, the metric did not stand up to further validity testing: while the metric was good at identifying individuals who genuinely did not wash their faces with soap and could accurately predict the proportion of individuals who did not wash their faces, it was much poorer at correctly identifying those who used soap. The very low positive predictive value (<15% for all but one indicator) means that most of the individuals identified as having washed their face with soap according to pictorial recall were not observed to have washed their face with soap. In other words, although the prevalence estimates for face washing with soap obtained through structured observation and pictorial recall were comparable, the two methods actually recorded different individuals as having washed their faces with soap. If soap availability in households truly varies across the week as reported by study participants, as pictorial recall collected data on the day prior to observation, we need to consider whether the specific individuals using soap genuinely differed between the methods. Presence of soap was verified during consenting (the reporting period for pictorial recall) and all households had bar and/or boxes of powdered soap; no households had single use soap sachets. Furthermore, many individuals observed to use soap during face washing did not report washing their face with soap during pictorial recall even though they had soap in the home. We consequently have good reason to believe that differences between the methods are due to reporting and not soap availability. If the prevalence of face washing with soap was higher, the low sensitivity suggests that pictorial recall would likely underestimate the true prevalence of face washing with soap. This study suggests that pictorial recall is not a valid alternative to structured observation for the measurement of face washing with soap and reflects knowledge rather than behaviour. This conclusion is further supported by scrutiny of the pictorial recall data, which showed clear measurement reactivity in the estimates of face washing with soap, which were inflated in the intervention arm at follow-up, but not at baseline.

Children aged 7–12 who washed their face with water during observation were significantly less likely to have a clean face than those who did not wash their face, but no other significant associations were observed between recent face washing and facial cleanliness. If bias had been introduced into the qualitative assessment of facial cleanliness due to knowing each child’s face washing status, we would expect the results to have been reversed. These findings are in line with studies that suggest that a clean face is not a good predictor of recent face washing with water [38, 39] and should not be used to measure face washing behaviour [38, 39]. However, face washing was not associated with improvements in qPHAT scores in our study either. The complete lack of association between face washing behaviour and face wipe dirtiness in our study is surprising, and contradicts findings from a recently published study which suggests that both observed oculo-nasal discharge and face wipe dirtiness scores are associated with face washing behaviour [41].

Our findings are most likely explained by a combination of a lack of effectiveness of face washing in this setting (only 3% of children washed with soap), the length of time between face washing and the facial cleanliness assessments (almost 3 hours on average) and the study context. Trends for the youngest children (whose faces are washed for them) and older children who wash their own faces suggest use of soap may remove more ocular discharge when faces are washed thoroughly, but the small sample size limits the conclusions that can be drawn from these analyses. Washing with water may be cursory or serve solely to refresh the face, perceived and implemented differently to face washing with soap [50]. West and colleagues do not report on water availability or the frequency of soap use during face washing in their study [41], but face washing may have been more thorough (effective) in their setting. As we were unable to measure facial cleanliness immediately after face washing due to the need to avoid biasing other data collection, it is not possible to know the extent to which face washing removed dirt and discharge from faces. The Tanzania study found face washing could be detected by observed discharge and face wipe dirtiness metrics up to 4 hours after washing [41], but qPHAT scores in our study did not improve even when there was a short lag time (< = 1.5 hours) between face washing and application of qPHAT. As active trachoma is associated with ocular discharge [46], and discharge has been demonstrated to return to faces after washing [38, 65], it is possible that faces became dirty more quickly in our high trachoma prevalence (and infection) study setting in Ethiopia than they did in Tanzania. It is nevertheless perplexing that washed faces had more discharge than unwashed faces. Real and perceived water insecurity in our study setting may impact decisions around face washing frequency and thoroughness. If water is scarce, it may be that children who produce more discharge are more likely to have their faces washed. This could make these faces appear dirtier if discharge returns over the course of the morning [47]. As we did not assess presence of oculo-nasal discharge at the start of the observation period (to avoid introducing bias), we cannot confirm this. Our study does not confirm the validity of facial cleanliness metrics for the assessment of face washing behaviour shown in Tanzania [41]. The use of facial cleanliness metrics as a proxy for face washing behaviour warrants further exploration prior to application at scale.

### Limitations

Our study had two key limitations. First, we used observation as the ‘gold standard’ for comparison with the other metrics. Although we can document whether a face was washed during the three-hour observation period and we made every effort to commence observation at dawn, before households awoke, due to logistical challenges getting 12 enumerators into place by dawn in a very rural setting, some observation sessions began after 6am. If face washing took place before the observation period commenced or away from the home, survey report and pictorial recall could be more accurate at estimating face washing prevalence than our results suggest. We have several reasons to believe that most face washes were captured by this study and differences between observation and alternative metrics were mainly due to reporting. First, review of the timing of face washes in households where observation began on time shows that few face washes would have been missed if the observation session had begun later. Second, sensitivity analysis of caregivers who were not observed to wash their faces found stratifying by observation start time did not change pictorial recall findings. Third, water was collected by only 1 in 5 households during observation, providing limited opportunity for face washing outside the home, as observed in our earlier study [50]. Finally, in this previous study in a similar setting in Oromia we observed behaviour overnight in nine households and we found that only 4% of early morning (pre 9am) faces washes took place before 6am. These early morning ablutions were all performed by adults. Whilst we cannot discount the possibility that the true prevalence of face washing may be slightly higher than captured through structured observation, we have confidence that the vast majority of face washes were recorded and the conclusions of this study are valid.

Second, structured observation recorded behaviour between 6 and 9 am, whilst survey respondents reported whether their face had been washed on the previous day “in the morning, shortly after waking” (details captured through follow up questions), and script-based pictorial recall captured behaviour chronologically from the time of waking, with the first segment of the day defined as the time from waking until midmorning (around 11am). Due to these differences in timing, measured differences in face washing prevalence between observation and the other metrics should not be considered absolute.

If we had been able to collect data on the cleanliness of children’s faces immediately after face washing, we would have been able to say more about the effectiveness of face washing in this study and the speed at which faces become dirty again in this setting. However, this was not possible due to the need to avoid introducing bias to the collection of structured observation, survey report and pictorial recall data. Results from the FAWASH trial that documents the presence of *Chlamydia trachomatis* bacteria, oculo-nasal discharge and qPHAT scores immediately prior to and following face washing and at intervals up to 8-hours after face washing are forthcoming (trial registration number ISRCTN 12814010).

## Conclusions

Survey (self)-report and script-based pictorial recall in this study were found to be inaccurate and we cannot recommend using these methods to measure face washing behaviour in community settings. It seems that even when done only in a small sample in a study population, structured observation is likely to provide greater insight than other, possibly more scalable methods using larger sample sizes, particularly in the context of a face washing behaviour change intervention. Caution should be applied before using facial cleanliness metrics as a proxy for face washing. Improving our ability to determine whether face washing campaigns have changed behaviour is crucial in the context of trachoma elimination strategies.
